# Applications of Theranostics for Detecting and Targeting CNS Injuries and Diseases

**DOI:** 10.1155/2022/9891859

**Published:** 2022-04-15

**Authors:** Yu-Yo Sun, Horacio Soto, Chung-Feng Kao, Cui Mei, Muh-Shi Lin

**Affiliations:** ^1^Institute of Biopharmaceutical Sciences, National Sun Yat-sen University, Kaohsiung 804201, Taiwan; ^2^Department of Neuroscience, Center for Brain Immunology and Glia (BIG), University of Virginia, School of Medicine, Charlottesville, VA, USA; ^3^Cancer Clinical Trials Access Program, Kaiser Permanente, Bellflower, USA; ^4^Department of Agronomy, College of Agriculture and Natural Resources, National Chung Hsing University, Taichung, Taiwan; ^5^Advanced Plant Biotechnology Center, National Chung Hsing University, Taichung, Taiwan; ^6^Department of Neurology, Huashan Hospital, Fudan University, Shanghai, China; ^7^Division of Neurosurgery, Department of Surgery, Kuang Tien General Hospital, Taichung, Taiwan; ^8^Department of Biotechnology and Animal Science, College of Bioresources, National Ilan University, Yilan, Taiwan; ^9^Department of Biotechnology, College of Medical and Health Care, Hung Kuang University, Taichung, Taiwan; ^10^Department of Health Business Administration, College of Medical and Health Care, Hung Kuang University, Taichung, Taiwan

As of today, the limit of medical science is the inability to efficiently and completely eliminate neuronal damage caused by secondary insult from neurological trauma and neurodegenerative diseases. In general, the primary insult occurs at the time of impact; it involves contusions, lacerations, and axonal injury as a result of shearing, tearing, or stretching with consequent impairment of the neural architecture. Initial traumatic injuries or pathogen-associated molecular patterns (PAMPs) from misfolded proteins evoke secondary insult by virtue of dynamic interactions among ischemic, inflammatory, cytotoxic processes, and mitochondrial dysfunction [1]. These types of molecular-level elements that may contribute to the holistic scale are implicated in the pathogenesis of neuro-oncology. Impaired immune competence, such as dysregulation of macrophages and microglia, and mitochondrial dysfunction reciprocally impact the microenvironment of glioblastoma and its prognosis [2]. As such, the future perspectives are to ensure the detection of disease at an extremely early stage through advanced diagnostic methods or to salvage primary lesions via advanced surgical interventions and, most fundamentally, to prevent widespread secondary injuries by exploring the microscopic pathogenesis or through interventions on a molecular level, which will be the objective and mission of the scientific community in the coming years.

Following a meticulous peer review process, we have decided to include 12 manuscripts in this special issue, composed of 11 research and 1 review article. In the clinical context, olfactory impairment is being recognized as a marker for the early detection of cognitive decline and Alzheimer's disease (AD) dementia. The transport pathways of olfactory signals superimpose substantially with those of dopamine and 5-hydroxytryptamine. Structural and physical dysfunction between the brain areas involved leads to dysregulated neurotransmission and accordingly to impaired olfaction [3]. Beyond olfactory dysfunction, X. Mei et al. indicated that amyloid *β* (A*β*) levels in the mouse retina corresponded to A*β* amounts in the brain; the appropriate time to measure retinal A*β* deserves emphasis. Moreover, Professor A. C. W. Huang et al. found in posttraumatic stress disorder (PTSD) animals a subtle affiliation of brain-derived neurotrophic factor (BDNF) expression in the medial prefrontal cortex (mPFC), amygdala, and hippocampus during situational reminders, whereas fluoxetine, a class of specific 5-hydroxytryptamine reuptake inhibitor drugs, was effective only on the basal amygdale, but not on the mPFC. The interconnections and pathogenesis of these complex neural structures need to be further understood in the setting of neurodegenerative diseases.

Early and nuanced clinical neurological symptoms can facilitate detection of disease, and subsequent remission of these neurological manifestations may even lead to greater postoperative benefit. Z. Wu et al. showed gait profile can aid in the diagnosis of early Parkinson's disease (PD). C.-L. Chen et al. reported that vascular cognitive impairment (VCI) with visual hallucinations frequently exhibited more severe dementia and neuropsychiatric symptoms. W. Lin et al. highlighted that more advantage can be gained through deep brain stimulation (DBS) under the proper treatment of neurological presentations during PD.

The tendency towards minimally invasive approaches or milder modalities has contributed to an improved quality of treatment. K.-Y. Chen et al. suggested that minimally invasive endoscopic-assisted surgery can be employed safely and effectively in the treatment of thalamic hemorrhage. T.-T. Chung et al. demonstrated that the smart antisnore pillow can be a useful device for patients with obstructive sleep apnea syndrome, rather than uncomfortable continuous positive airway pressure.

The implementation of artificial intelligence technology assists in early detection, shortens the period of diagnosis, and forecasts the outcome of the disease. Z.-Q. Pan et al. display that machine learning-based radiomic features (tumour shape, intensity, and texture) can predict the response to radiotherapy in patients with glioblastomas. Professor J. Jiang et al. introduced a metabolic connectome-based predictive model for ^18^F-fluorodeoxyglucose- (FDG-) PET images to facilitate the identification of brain metabolic dysfunction and established a clinically applicable biomarker to predict the progression of mild cognitive impairment to AD.

Upstream blockage of molecular pathogenic mechanisms contributes to the repression of disease advancement. In their review, T. Pi et al. underlined that the environmental factor, homocysteine, has mediated DNA methylation and contributes to the development of AD. CDGSH iron-sulfur structural domain 2 (CISD2) exerted anti-inflammatory effects by functioning as an upstream regulatory constituent of the PPAR-*β*/NF-*κ*B signal through the findings of M.-S. Lin et al. The anti-inflammatory natural compound wild bitter melon (WBM) showed a modulatory effect on CISD2 expression in injured animals and cells. This protective protein, CISD2, holds high potential as a target for the treatment of injury and disease in the central nervous system (CNS) [4].

Ultimately, we expect the endeavors of the authors to generate multidisciplinary viewpoints and novel insights that will achieve further advances in the domain of CNS injury and disease.

*Yu-Yo Sun* *Horacio Soto* *Chung-Feng Kao* *Cui Mei* *Muh-Shi Lin*

## References

[1] Lee S., Park S., Won J., Lee S. R., Chang K. T., Hong Y. (2015). The incremental induction of neuroprotective properties by multiple therapeutic strategies for primary and secondary neural injury. *International Journal of Molecular Sciences*.

[2] Lu R. O., Ho W. S. (2020). Mitochondrial dysfunction, macrophage, and microglia in brain cancer. *Frontiers in Cell and Development Biology*.

[3] Lin M. S., Chiu I. H., Lin C. C. (2021). Ultrarapid inflammation of the olfactory bulb after spinal cord injury: protective effects of the granulocyte colony-stimulating factor on early neurodegeneration in the brain. *Frontiers in Aging Neuroscience*.

[4] Lin M. S. (2020). CISD2 attenuates inflammation and regulates microglia polarization in EOC microglial cells-as a potential therapeutic target for neurodegenerative dementia. *Frontiers in Aging Neuroscience*.

